# Protein language models meet reduced amino acid alphabets

**DOI:** 10.1093/bioinformatics/btae061

**Published:** 2024-02-03

**Authors:** Ioan Ieremie, Rob M Ewing, Mahesan Niranjan

**Affiliations:** Vision, Learning & Control Group, University of Southampton, Southampton SO17 1BJ, United Kingdom; Biological Sciences, University of Southampton, Southampton SO17 1BJ, United Kingdom; Vision, Learning & Control Group, University of Southampton, Southampton SO17 1BJ, United Kingdom

## Abstract

**Motivation:**

Protein language models (PLMs), which borrowed ideas for modelling and inference from natural language processing, have demonstrated the ability to extract meaningful representations in an unsupervised way. This led to significant performance improvement in several downstream tasks. Clustering amino acids based on their physical–chemical properties to achieve reduced alphabets has been of interest in past research, but their application to PLMs or folding models is unexplored.

**Results:**

Here, we investigate the efficacy of PLMs trained on reduced amino acid alphabets in capturing evolutionary information, and we explore how the loss of protein sequence information impacts learned representations and downstream task performance. Our empirical work shows that PLMs trained on the full alphabet and a large number of sequences capture fine details that are lost in alphabet reduction methods. We further show the ability of a structure prediction model(ESMFold) to fold CASP14 protein sequences translated using a reduced alphabet. For 10 proteins out of the 50 targets, reduced alphabets improve structural predictions with LDDT-Cα differences of up to 19%.

**Availability and implementation:**

Trained models and code are available at github.com/Ieremie/reduced-alph-PLM.

## 1 Introduction

Protein sequence databases and their tools are the main drive to molecular biology research and analysis, but the rapid growth of protein sequence data allowed the introduction of deep learning models borrowed from natural language processing to bioinformatics tasks. Most notably, the scaling of unsupervised training on large language models allows the prediction of protein structures from single protein sequences (Lin *et al.* 2022). Language models set proxy tasks where the masked token (Devlin *et al.* 2019) is corrupted and then reconstructed in order to extract deep contextualized representations of the input and improve performance on downstream tasks. Reduced amino acid alphabets have been developed in the past, but their application to protein language models (PLMs) is unexplored. This raises the question of whether a more simplified encoding of protein sequences allows PLMs to learn the same patterns.

Amino acid alphabet reduction methods are of particular interest due to their ability to simplify the protein sequence space while still capturing structural information about proteins. Early mutation experiments (Heinz *et al.* 1992) on the T4 lysozyme protein showed a high degree of redundancy present at the sequence level. Similarly, the SH3 domain (small β protein) could be reconstructed by using only a five-letter alphabet: Ile, Lys(K), Glu(E), Ala, Gly (Riddle *et al.* 1997).

The simplest amino acid alphabet reduction is the HP model (hydrophobic, polar) which only encapsulates the concept of hydrophobic interactions. A less radical reduction was proposed by Wang (Wang and Wang 1999) by minimizing the mismatch within the MJ contact potentials matrix. The resulting reduced alphabet of five letters [similar to (Riddle *et al.* 1997)] used to encode protein sequences showed good performance for successful folding. What followed is several numbers of proposed alphabet reduction schemes that are applied to sequence alignment and protein folding, but with the majority not being applied to specific research work (Liang *et al.* 2022).

We focus here instead on a small subset of alphabets with various sizes that are popular in the literature. Apart from the WWMJ5 alphabet (Wang and Wang 1999), we use the GBMR4 and GBMR7 alphabets, designed by maximizing the mutual information between the reduced sequence and structural information (Solis and Rackovsky 2000). SDM12 and HSDM17 were developed from structurally determined substitution matrices and the MMSEQS12 alphabet was developed for fast clustering of protein sequences (Steinegger and Söding 2018). We introduce two extra alphabets based on hydrophobicity and learned clusters from a trained PLM using the full alphabet. Alphabets and their clusters based on 1-letter residue names are displayed in Table 1.

**Table 1.** Amino acid alphabets and their clusters.^a^

Alphabet	Clustering
UNIPROT20	A R N D C Q E G H I L K M F P S T W Y V
UNIPROT18	A R N D C Q EP G HL I K M F S T W Y V
HSDM17	A D KE R N T S Q Y F LIV M C W H G P
MMSEQS12	AST LM IV KR EQ ND FY C G H P W
WASS14	WM DI P C AV K T RE G L Y SH F NQ
SDM12	A D KER N TSQ YF LIVM C W H G P
GBMR7	DN AEFIKLMQRVWY CH T S G P
WWMJ5	CMFILVWY ATH GP DE SNQRK
GBMR4	ADKERNTSQ YFLIVMCWH G P

a Alphabet names are based on their literature abbreviations followed by a number denoting the number of clusters.

Here, we pre-train PLMs from scratch with different encodings imposed by the amino acid alphabets, and we investigate the performance on a set of downstream tasks: enzyme classification, homology detection, and three protein engineering datasets. Subsequently, we analyze ESMfold, a recent single-sequence protein folding model, in its capacity to predict the structures of protein sequences from the CASP14 target set (Kryshtafovych *et al.* 2021). The employed proteins undergo a process of translation through reduced alphabet reduction schemes before being presented as input to the folding model.

## 2 System and methods

### 2.1 Datasets

**Uniref**90**:** The pretraining dataset is composed of 76 215 872 protein sequences retrieved from Uniref (Suzek *et al.* 2007), with a sequence identity of <90%. The retrieval date is July 2018. For the non-standard amino acids selenocysteine (U) and pyrrolysine (O), we encode them as cysteine (C) and lysine (K). The remaining B, Z, X are all labelled as unknown (X). The final alphabet is of size 21, with 20 standard amino acids and 1 extra for the unknown type.

**Enzyme classification:** The enzyme dataset used for evaluation consists of 29 215 protein chains for training, 2562 protein chains for validation, and 5651 protein chains for testing, proposed by Hermosilla Casajús *et al.* (2021) and Dana *et al.* (2019). These proteins can be classified into 384 enzyme classes. The evaluation metric used is accuracy, and the reported results are based on the test set. To ensure data integrity and prevent data leakage, the dataset is further filtered (see Supplementary Material).

**Remote homology detection:** The remote homology dataset assesses the PLM’s ability to detect structural similarities across distantly related proteins. The evaluation metric is accuracy, and the task involves predicting the correct fold at three levels: fold, superfamily, and family. The dataset consists of 16 712 proteins belonging to 1195 folds (Rao *et al.* 2019).

**Protein engineering datasets:** The PLM’s performance is also evaluated on three protein engineering datasets (Dallago *et al.* 2021). These datasets focus on assessing the model’s ability to capture the effects of mutations and predict fitness and thermostability. *GB1 Landscape*: This dataset involves predicting the binding fitness changes due to mutations in the GB1 protein domain (Wu *et al.* 2016). *AAV Mutational Screening Landscape*: The dataset measures the ability to predict fitness changes in the context of adeno-associated viruses (AAVs) (Bryant *et al.* 2021). *Meltome Atlas*: The dataset focuses on predicting thermostability of proteins (Jarzab *et al.* 2020). We assessed performance using various approaches: training on single mutants and testing on the rest (‘1-versus-many’), training on single and double mutants and testing on the rest (‘2-versus-many’), and training on mutants with up to three/seven changes and testing on the rest (‘3/7-versus-many’). Additionally, we trained on sequences with low fitness values (lower than wild type) and tested on sequences with high fitness values (‘low-versus-high’). For the meltome dataset, results are presented on a mixed split, where test sequences have only 20% sequence identity to the training set.

**CASP14:** For analyzing the effect of alphabet reduction on structure prediction, we use the 51 test targets proposed in CASP14 (Kryshtafovych *et al.* 2021). We remove the target T1044 due to its long sequence, which cannot be modelled on a consumer GPU.

### 2.2 Protein language models

We employ a three-layer BiLSTM similar to previous work (Bepler and Berger 2018, Alley *et al.* 2019) trained on masked token prediction. We use a smaller architecture compared to recent transformer models (14M versus 650M parameters of ESM) (Rives *et al.* 2021) to allow the pre-training of multiple models. However, the smaller model is powerful enough to capture evolutionary information useful for various prediction tasks (Alley *et al.* 2019). Each amino acid is first passed through a learned embedding layer to allow projecting them into 2D for visualization. Each model is trained using Distributed Parallel (Paszke *et al.* 2019) on multiple GPUs with a total batch size of 1024 sequences. We use a learning rate of 2e–4 with a linear weight decay up to a tenth of the initial value and the Adam optimizer with the default Pytorch parameters. The model is trained to recover masked residues using the cross-entropy loss. We apply a residue masking strategy with a 10% probability. Rather than utilizing a designated mask token, we substitute the masked residues with tokens from other residues. These replacement tokens are selected based on a background distribution calculated using Uniref90. Each model took around 6 days to train for 240*k* steps, which is the equivalent of 3.3 passes through Uniref90.

We use the ESMfold model (version 0, 3B parameters) (Lin *et al.* 2022) to fold protein sequences. This is a version trained on data with a cut-off date before the CASP14 competition.

### 2.3 Protein sequence translation

**Figure 1.**
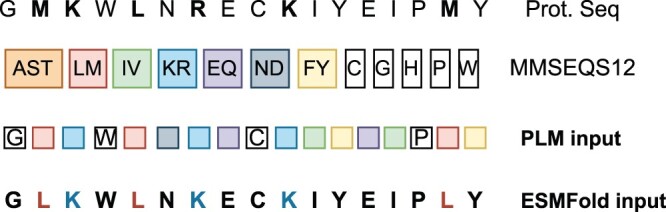
A protein coding representation using the MMSEQS12 alphabet. Trained PLMs consume the sequence as a token representation (cluster colour). ESMFold gets as input a protein sequence where a representative amino acid is chosen from each cluster at random and kept constant along the sequence. For example, amino acids Leu(L) and Met(M) are all changed to Leu as they belong to the same cluster.

For the PLMs, converting protein sequences into a reduced alphabet involves assigning a single token to all residues within a specific cluster. For instance, in the MMSEQS12 alphabet, the residues Ala, Ser, and Thr are all represented by the same token (see Fig. 1). The model still processes the same 21D vector for each residue in the sequence, but some dimensions do not code for anything.

For protein folding, we need to select a residue letter from each cluster as ESMfold needs a protein sequence as input. A representative residue is randomly chosen from the cluster to stand in for all other residues and kept fixed along the input sequence. We explore all possible ways of encoding sequences this way along with the option to randomly select a residue at each location in the Supplementary Material. We compute the identity and similarity values between the translated sequences and the original full alphabet sequences for the CASP14 dataset using Blosum62 (see Supplementary Material).

## 3 Results and discussion

### 3.1 Performance of PLMs on downstream tasks

PLMs are faced with a difficult task: reconstructing missing parts of the sequence space without any knowledge of structural or functional constraints imposed during evolution or physico-chemical characteristics of each amino acid. However, perplexity seems to be a good proxy measure of ‘understanding’ proteins and their structures, with models that perform better on the language task showing an increased ability to predict protein structures (Lin *et al.* 2022).

We estimate the perplexity of the language model using a random held-out validation dataset (1% of Uniref90). The perplexity for a protein sequence *x* is defined as the exponential of the negative log-likelihood and can be described as:

(1) $$\text{Perplexity}(x)=\text{exp}\;\{-\text{log}\;p(x_{i\in M}|x_{j\notin M})\}$$

The set *M* is a random variable and represents the set of masked tokens.

#### 3.1.1 PLMs learn a systematic clustering of the amino acids

**Figure 2.**
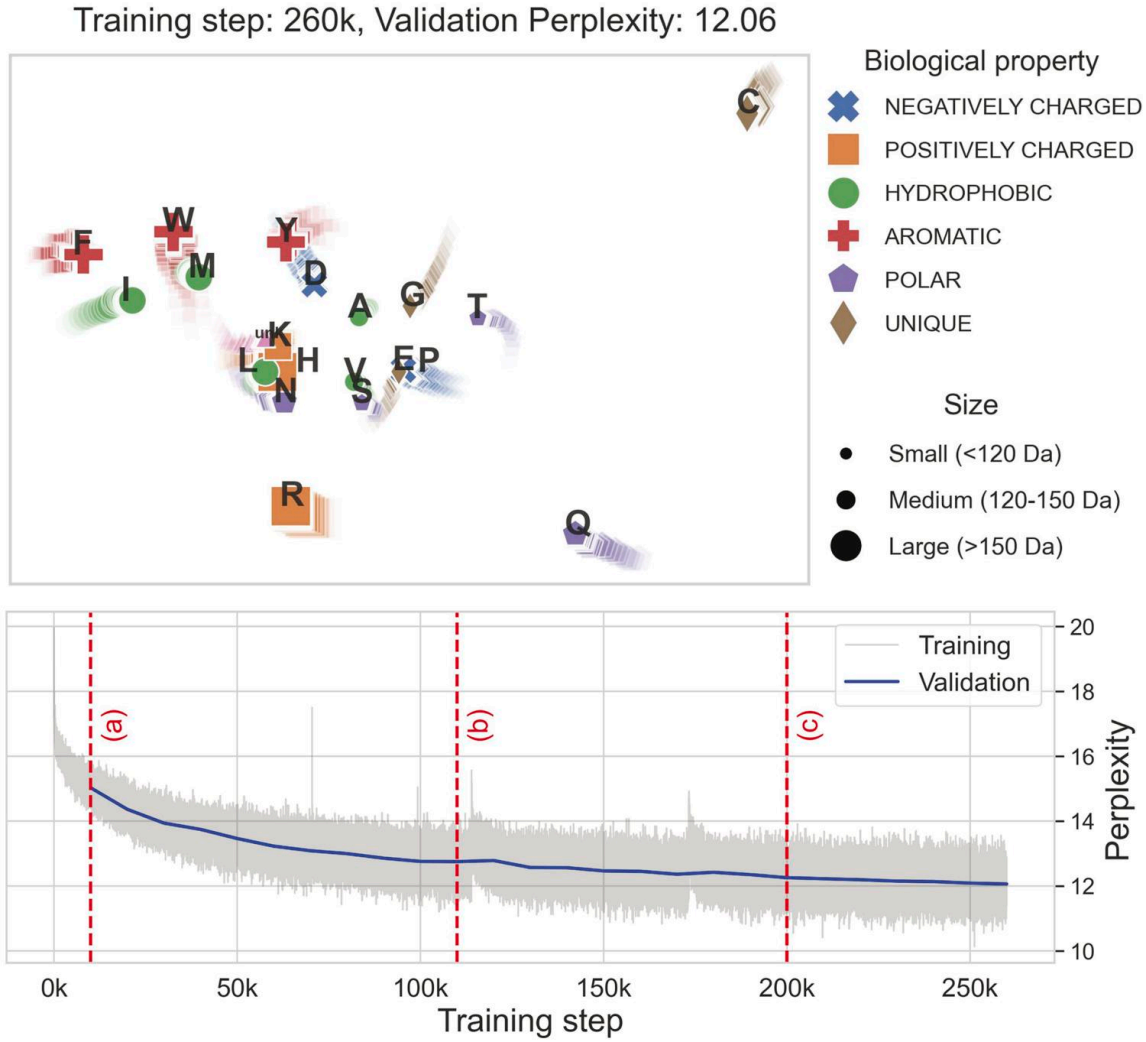
PCA projection of the amino acid embedding layer during training, along with the training and validation perplexity of the PLM trained on the full alphabet. Based on the embedding projections, we depict three clustering stages. During the first clustering stage (a), a clear improvement in perplexity is related to the separation of amino acids Cys, Arg(R), and Gln(Q). During stage (b), the language model clusters amino acids based on hydrophobicity (hydrophobic/polar), while in stage (c) amino acids are further grouped within their clusters. Amino acid pairs Pro-Glu and Leu-His are clustered together, which suggests strong similarities found by the language model.

To get a better insight into how the PLM starts differentiating between the 20 amino acids, we project the embedding layer into 2D using PCA at various points during training. In Fig. 2, we plot the perplexity of the model along with the projection of the amino acids during training (full animation: github.com/Ieremie/reduced-alph-PLM). In the first 10*k* training updates (a), a major improvement in perplexity is given by singling out amino acids Cys, Gln(Q), and Arg(R). Cystine has a unique solvent accessibility behaviour, as it is often found buried within the protein core and can form disulfide bonds. Gln and Arg are also singled out from any grouping, but their polar character or volume (Taylor 1986) is not unique and does not explain the separation. The second clustering stage (b) seems to be driven by hydrophobicity, with Phe(F), Trp(W), Ile, Met forming the hydrophobic cluster, Ser, Pro, Glu(E) forming the polar group and Ala, Gly and Thr forming the moderately hydrophobic cluster. The third stage (c), modifies the positions of amino acids within clusters, making the Pro-Glu and Leu-His amino acid pairs. After 200*k* steps, the improvement in the perplexity of the model is relatively low, with only slight changes in how amino acids are projected in 2D. We hypothesize that the model’s perplexity is mostly influenced by the ability to distinguish amino acids and to create embeddings that are powerful enough to create separation regardless of the sequence context.

Based on the clusters found by the language models, we create a new alphabet named UNIPROT18 that encodes the Pro-Glu and Leu-His amino acid pairs the same way. We reason that this simplification based on the model suggestions of similarity should be able to learn the same evolutionary information and perform the same on downstream tasks.

As most of the amino acids that are frequently clustered together are highly similar in terms of hydrophobicity (Liang *et al.* 2022), we develop a clustering method based on the solvent-accessible surface area, namely WASS14 (see Supplementary Material).

#### 3.1.2 PLMs trained on reduced AA alphabets capture meaningful representations

Encoding protein sequences based on predefined clusters compresses the input space such that sequences that only differ in terms of in-cluster assignments will be regarded as the same. This essentially reduces the sequence fidelity, which might allow the model to focus on learning more general representations that go beyond single-point mutations.

To evaluate if this is the case, we fine-tune the pre-trained language models on a set of downstream tasks. To go from a sequence of embeddings to a number of classes or a regression output, we pass the sequence of embeddings through an attention-pooling layer followed by an MLP with a single hidden layer. For the Fold task, we provide standard deviations over six runs with slightly different learning rates as the model tends to perform better on a specific split (e.g. superfamily) depending on the learning rate. Similarly, for the enzyme task, we report results from four runs.

In Table 2, it can be observed that the language model trained on the full alphabet performs better than any other encoding on both tasks. The model performance is similar to ESM-1B (Rives *et al.* 2021), with differences appearing only on the Superfamily and Family classification, where the scale of the model allows it to generalize better. However, the HSDM17 alphabet allows the trained PLM to capture enough information to perform similarly with the full alphabet across tasks. This suggests that PLM can still capture evolutionary information even if certain mutation pairs are never seen during pre-training. Similarly, the MMSEQS12 and SDM12 alphabets exhibit only a small loss of performance. UNIPROT18, a reduced alphabet generated by analyzing the clusters found by a PLM, exhibits lower performance across tasks compared to the full alphabet. This suggests that even if certain residues appear similar when analyzing their embeddings, removing even two mutation pairs from the pretraining dataset hinders the ability of language models to capture evolutionary information.

**Table 2.** PLMs performance (accuracy) on the Fold and Enzyme tasks.^a^

Alphabet	FOLD			REACT %
	Fold %	Super. %	Fam. %	
ESM-1b	26.8	60.1	97.8	83.1
**UNIPROT20**	**26.3** ± 0.96	**43.3** ± 0.41	**90.7** ± 0.44	**81.8** ± 0.39
**UNIPROT18**	24.2 ± 0.46	38.5 ± 0.93	86.5 ± 1.59	77.9 ± 0.80
**HSDM17**	**25.4** ± 1.00	**42.1** ± 0.59	**87.9** ± 1.25	80.6 ± 0.85
**MMSEQS12**	23.5 ± 0.91	39.4 ± 1.03	87.3 ± 0.93	**80.7** ± 0.69
**WASS14**	20.5 ± 0.55	28.9 ± 0.57	78.0 ± 0.94	75.4 ± 0.55
**SDM12**	23.8 ± 0.97	39.1 ± 0.84	85.5 ± 1.32	80.5 ± 0.92
**GBMR7**	15.5 ± 0.90	21.9 ± 0.70	67.7 ± 3.63	70.8 ± 1.12
**WWMJ5**	17.5 ± 1.00	25.4 ± 0.44	72.4 ± 1.47	73.8 ± 1.05
**GBMR4**	12.3 ± 0.89	11.4± 0.46	47.9 ± 2.43	68.3 ± 3.28

a The bold values represent the top two best-performing models excluding ESM-1b. Uncertainties for the folding task/enzyme task are standard deviations over 6/4 runs with different learning rates. The performance of ESM-1b is sourced from Zhang *et al.* (2022).

Surprisingly, the WASS14 alphabet based on hydrophobicity performs poorly across tasks. This might suggest that alphabets based purely on solvent accessibility throw away too much information. This is also suggested by the MMSEQS12 alphabet which has the same size, but a much smaller information loss.

The comparison on downstream tasks suggests that although PLM trained on reduced alphabets can retain some level of information, they are limited in capturing the full sequence diversity found within the Uniref database. Even on tasks involving low sequence identity such as the Fold classification, where reduced alphabets were expected to perform better by avoiding explicit mutations, they fall short compared to the full alphabet.

#### 3.1.3 Small alphabets distort evolutionary information

An immediate trend appearing in Table 2 is the performance degradation as alphabets get smaller. PLM trained with alphabets GBMR4, GBMR7, and WWMJ5 do not seem to offer useful embeddings for the two downstream tasks. This is similar to previous work (Murphy *et al.* 2000), where alphabets that have a size of <10 clusters degrade the sequence information in such a way that it does not allow the discovery of structural homologs.

As PLM are trained to understand sequence context, proteins encoded with highly reduced alphabets do not provide enough diversity to allow the learning of complex relations. For example, long-range dependencies due to residue co-evolution could be completely lost. This can also be observed during pretraining, where there is only a small improvement in terms of perplexity when it comes to training on alphabets with a few clusters.

#### 3.1.4 Reduced amino acid alphabets cannot capture the effects of mutations

**Figure 3.**
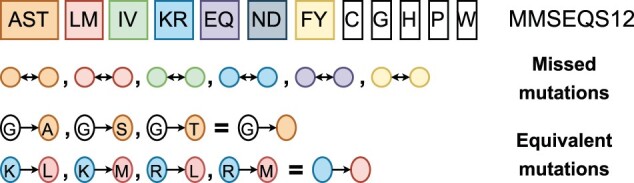
An illustration of how the MMSEQS12 alphabet captures the effect of mutations. Mutations appearing between residues of the same cluster are encoded the same way and cannot be captured (missed). Residues mutating to any cluster member share equivalent representations.

**Figure 4.**
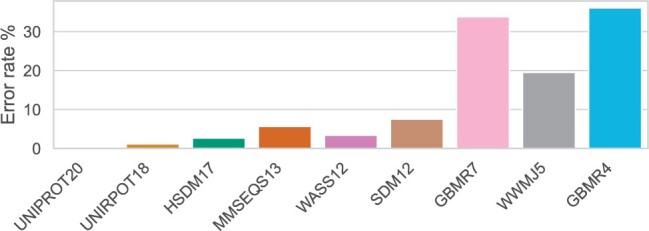
The error rate of the amino acid alphabets as the percentage of mutation pairs from the AAV dataset that cannot be distinguished. Alphabets with a few clusters are unable to capture the effect of mutations. Notice the low number of errors made by the WASS14 alphabet compared to alphabets of similar size due to its design based on hydrophobicity.

A major downside of using reduced alphabets is their inability to distinguish mutations that appear within clusters. For example, if Leu and His are encoded using the same token, any two sequences that differ in terms of Leu-His mutations will be regarded as identical (see Fig. 3). This is less likely to become a problem for fold recognition or enzyme classification, however for protein engineering where a single mutation can affect stability, this becomes a significant limitation. To exemplify this case, a set of amino acid substitutions is created using the AAV dataset from FLIP (Dallago *et al.* 2021). An error is an alphabet encoding that fails to distinguish a mutation from the wild type. Figure 4 shows that as alphabets get smaller, the error rate in distinguishing mutations from the wild type increases. The WASS14 alphabet, based on clustering together residues that have similar hydrophobicity profiles, has a low error rate compared to other alphabets of similar size. This might be due to a lower mutation rate between residues with no differences in their hydrophobic character.

To evaluate to what degree PLM trained on reduced alphabets can still perform on protein engineering tasks, we fine-tune the models on the GB1, AAV, and Meltome datasets. We find that the validation splits provided in the FLIP benchmark (Dallago *et al.* 2021) used for early stopping are the reason behind some poor runs. Therefore, we report the best performance metrics including the results from three extra seeds that use a random validation size of 30%. Table 3 highlights that the full alphabet performs comparably with the much larger CARP model (Yang *et al.* 2022) on most of the splits, which allows a fair comparison between PLMs trained on reduced alphabets. Surprisingly, alphabets with >10 clusters perform better than the full alphabet on certain splits. Namely, WASS14 on the ’2/7-versus-many AAV’ splits, and MMSEQS12 on the ’7-versus-many AAV’ split. This suggests that while the full alphabet has the ability to distinguish between all mutation pairs that appear in the dataset, in certain cases ignoring those mutations and focusing on out-of-clusters mutations improves performance.

**Table 3.** Performance (Pearson correlation) on the FLIP tasks.^a^

Alphabet	AAV				GB1			Meltome
	1-versus-many	2-versus-many	7-versus-many	low-versus-high	2-versus-many	3-versus-many	Low-versus-high	Mixed-split
CARP-640M	0.73 ± 0.05	0.81 ± 0.03	0.77 ± 0.03	0.19 ± 0.008	0.73 ± 0.03	0.87 ± 0.004	0.43 ± 0.04	0.53
**UNIPROT20**	**0.41** ± 0.08	0.48 ± 0.00	0.55 ± 0.06	**0.20** ± 0.04	**0.64** ± 0.03	**0.82** ± 0	**0.39** ± 0.09	**0.28**
**UNIPROT18**	0.40 ± 0.03	0.43 ± 0.05	0.56 ± 0.08	**0.20** ± 0.08	**0.64** ± 0.02	0.81 ± 0	0.27 ± 0.12	0.27
**HSDM17**	0.30 ± 0.07	0.44 ± 0.01	0.60 ± 0.04	**0.20** ± 0.04	0.61 ± 0.02	0.79 ± 0	0.26 ± 0.04	0.27
**MMSEQS12**	0.32 ± 0.06	0.45 ± 0.05	**0.62** ± 0.00	0.09 ± 0.03	0.53 ± 0.01	0.73 ± 0	0.20 ± 0	0.22
**WASS14**	0.35 ± 0.05	**0.51** ± 0.01	**0.62** ± 0.02	0.07 ± 0.07	0.46 ± 0.07	0.73 ± 0.01	0.13 ± 0.06	0.24
**SDM12**	0.40 ± 0.03	0.36 ± 0.04	0.59 ± 0.01	0.08 ± 0.03	0.61 ± 0.04	0.76 ± 0	0.33 ± 0.02	0.24
**GBMR7**	0.34 ± 0.01	0.26 ± 0.06	0.47 ± 0.06	0 ± 0.02	0.41 ± 0.02	0.49 ± 0	0 ± 0.01	0.20
**WWMJ5**	0.31 ± 0.04	0.43 ± 0.02	0.56 ± 0	0.01 ± 0.01	0.44 ± 0.03	0.60 ± 0.01	0.10 ± 0.01	0.25
**GBMR4**	0.25 ± 0.01	0.38 ± 0.03	0.46 ± 0.01	0 ± 0.02	0.41 ± 0.02	0.52 ± 0	0.05 ± 0	0.21

a Uncertainties are standard deviations over three seeds. For the Meltome dataset, we train a single model to reduce the computational cost. We include the performance of CARP-640M for reference (Yang *et al.* 2022) and mark as bold the best model excluding CARP-640M.

### 3.2 ESMfold with compressed protein sequences

In the previous section, we highlighted the downsides of training a PLM on a training set where certain mutation pairs are never modelled for. Here, we explore the ability of a large language model to predict the structures of a set of proteins, given that their sequences have been simplified using different alphabet reduction schemes. We use ESMfold (version 0, 3*B* parameters, Lin *et al.* 2022) instead of Alphafold (Jumper *et al.* 2021b) as it is a single sequence folding model that does not rely on the creation of MSAs. This would ensure that a good prediction is solely based on the input sequence and not the ability of multiple sequence alignment tools to find similar sequences. The underlying idea is that a model faced with a sequence of lower complexity can act as a sequence template for a multitude of protein sequences that code for the same protein fold (Li *et al.* 1996). A folding model faced with such a sequence can design the structure without the constraints of specific evolutionary information tied to a particular protein of interest. To test this aspect, we fold the protein sequences used as targets in the CASP14 competition. We translate each protein sequence using the alphabets mentioned above and use them as inputs to ESMfold.

To evaluate the prediction accuracy, we calculate the LDDT-Cα scores (Local Distance Difference Test) relative to the CASP14 targets (Mariani *et al.* 2013). This metric is computed solely on the backbone of the structure, similar to previous work (Jumper *et al.* 2021b), and it represents a superposition-free score. It is important to note that ESMfold takes the entire domain sequence context as input, while the target we use for scoring is a segment within the predicted structures. To account for this, we crop the predicted structures according to the domain range specified in CASP14.

**Figure 5.**
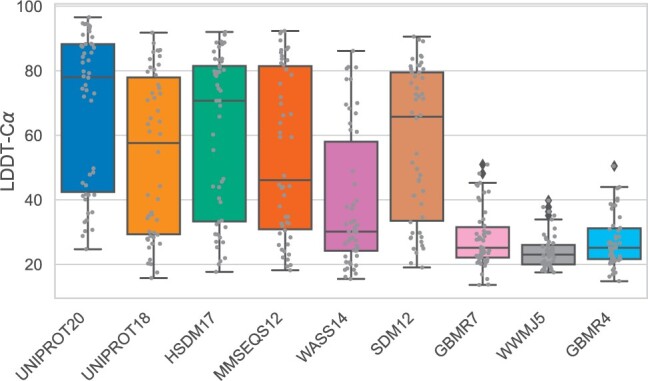
The LDDT-Cα values for the CASP14 targets, predictions made by ESMfold using different sequences. Each grey point represents a protein in the target set. Alphabets with <10 clusters generate predicted structures with low LDDT-Cα scores. The alphabet SDM12 allows the prediction of high-quality protein models by only using 12 residues to encode the sequence.

Figure 5 presents box plots illustrating the LDDT-Cα values of the predicted structures across various alphabet reduction methods. When the original sequence is employed as input (UNIPROT20), the majority of the predicted structures emerge as favourable decoys, showcasing high LDDT-Cα scores. The HSDM17 alphabet outperforms UNIPROT18, despite its smaller size, and delivers results similar to those achieved with the complete alphabet. SDM12 maintains a robust LDDT-Cα mean even after omitting 8 residue letters from the input sequence. This suggests that ESMfold retains its ability to comprehend these protein sequences, capturing residue interactions through generalization to homologous sequences encountered during training. On the other hand, small alphabets (<10 clusters) yield low LDDT-Cα scores (<50) for all targets. This signifies that in the majority of cases, small alphabets tend to distort the underlying meaning of the protein sequence, which cannot be reliably understood by the folding model.

**Figure 6.**
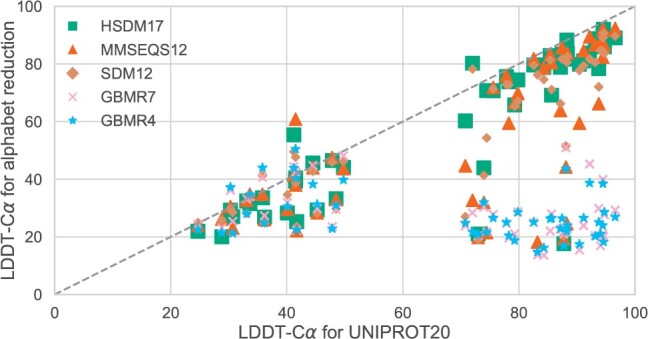
A direct comparison shows the LDDT-Cα score differences obtained from the original sequence and its translated version. Each data point corresponds to a target within the CASP14 dataset. Notably, both HSDM17 and SDM12 demonstrate LDDT-Cα values comparable to the full alphabet across the dataset. Note the presence of proteins for which predicted structures improve with the use of alphabet reduction methods. Large improvements are observed in proteins for which the original sequence prediction is far from the target structure (LDDT-Cα < 50).

In Fig. 6, a direct comparison of the LDDT-Cα scores for each target is presented across various alphabet encodings. Notably, larger alphabets, such as HSDM17, exhibit performance similar to UNIPROT20 across the entire LDDT-Cα range. Conversely, smaller alphabets yield diminished performance for targets that demonstrate high-accuracy predictions when utilizing the original sequence. However, these smaller alphabets align with the scores of the full alphabet for targets that are difficult to predict (LDDT-Cα < 40).

**Figure 7.**
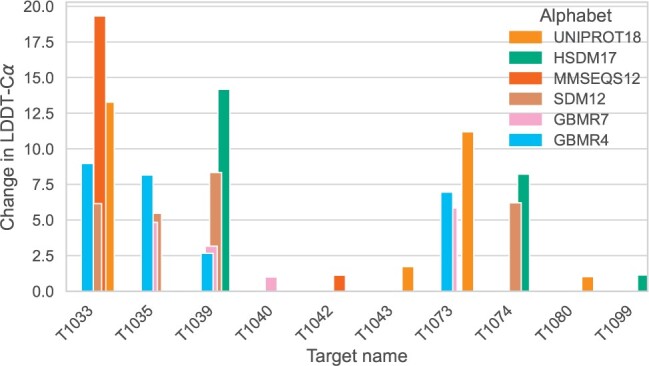
Ten targets from CASP14 for which the alphabet reduction methods improve the overall LDDT-Cα scores by a margin of at least 1%. The target T1039, which belongs to the FM (free modelling) category, is better predicted by ESMfold when using the HSDM17 alphabet, with an increase of LDDT-Cα of 14%. Similarly, for the T1033 target, the difference in LDDT-Cα score to the original sequence is 19%. For the target T1035, small alphabets show improvements in LDDT-Cα over 5%.

**Figure 8.**
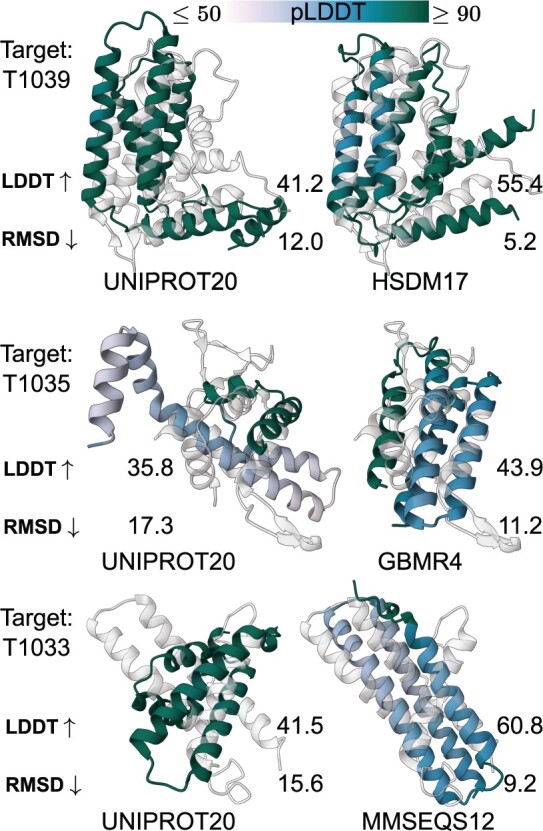
Structural predictions for the T1039, T1035, and T1033 targets using different alphabets to encode the input sequence. The structure in light grey represents the ground truth, while the superimposed one is the ESMfold prediction, coloured by the predicted LDDT (confidence). HSDM17, GBMR4, and MMSEQS12 alphabets aid the prediction of more accurate decoys.

Of particular interest are the instances where using a reduced alphabet leads to improved LDDT-Cα scores. These improvements are relatively modest for targets that are predicted with high accuracy using the full alphabet. It is the predictions with low accuracy that exhibit the most substantial improvement. A comprehensive view of the overall improvement for these targets can be found in Fig. 7. In Fig. 8, the structures and the ESMfold predictions for targets T1039, T1035, and T1039 are superimposed. We selected them for visualization due to their high improvements in structure prediction. Predictions made using the modified sequences are labelled as less confident (low pLDDT-Cα), but end up being more structurally similar to the target structure. The target T1035, translated using the GBMR4 alphabet is only 31% similar to the original sequence. However, this highly modified sequence is folded into a protein structure that improves LDDT-Cα scores of up to 7.5%.

Targets T1039 and T1033 display the highest gain in performance when ESMfold gets as input a sequence translated using a reduced alphabet. It is intriguing, however, that by directly hindering the information contained within the protein sequence, a more accurate prediction can be generated. These targets belong to the FM-free modelling category, meaning that at the time of the competition, no structural templates were available. Having a closer look at AlphaFold’s approach to these targets (Jumper *et al.* 2021a), it becomes evident that these domain-level targets possessed shallow MSAs. AlphaFold employed instead a ‘crop-then-fold’ method to improve predictions. This entails the creation of larger MSAs by using the full protein sequence (T1044), and then cropping the MSAs for the domains of interest. This method was shown to improve the predictions of the domains T1039 and T1033.

**Figure 9.**
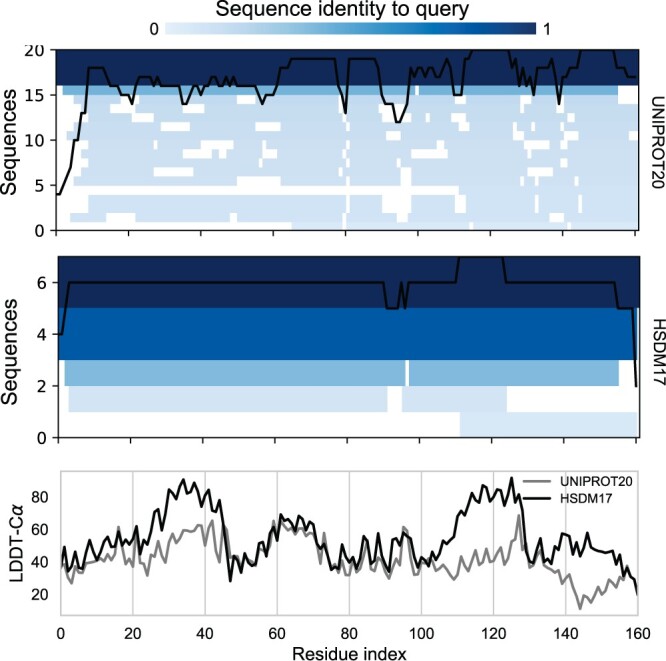
The first two plots show the multiple sequence alignments for the target T1039: the first one uses the original sequence as a query while the second one uses the modified one from the HSDM17 alphabet. Note the smaller MSA size when using the modified sequence. On the bottom, LDDT-Cα values for the predicted structures are plotted along the chain. The predicted structure using the HSDM17 alphabet has regions with higher LDDT-Cα values, but these are not due to the MSA depth.

To determine whether the enhanced accuracy stems from ESMfold’s capability to tap into a more extensive MSA during inference, we visualize the MSAs obtained from the original and modified sequences of target T1039. We use the MSA generation method from ColabFold (Mirdita *et al.* 2022). In Fig. 9, it becomes apparent that the MSA of the modified sequence is much shallower. The prediction made using the HSDM17 alphabet has regions with higher LDDT-Cα values, but these are not due to a deeper region of the multiple sequence alignment. Conversely, the scale of the language model is the main driver of accurate predictions by improving sequence perplexity (Lin *et al.* 2022). This entails that an input sequence with a reduced perplexity could enable the generalization of the model to a wider number of sequences that fit the sequence template. To test this aspect, we compute the pseudo-perplexity for each target. This computation involves employing L-forward passes with mask token prediction, to calculate the likelihood of each residue at every position. This is a more robust measure of a sequence perplexity compared to Equation 1 as it is deterministic.

(2) $$\text{PPPL}(x)=\text{exp}\;\{-\frac{1}{L}\text{log}\;p(x_{i}|x_{\neg i})\}$$

**Figure 10.**
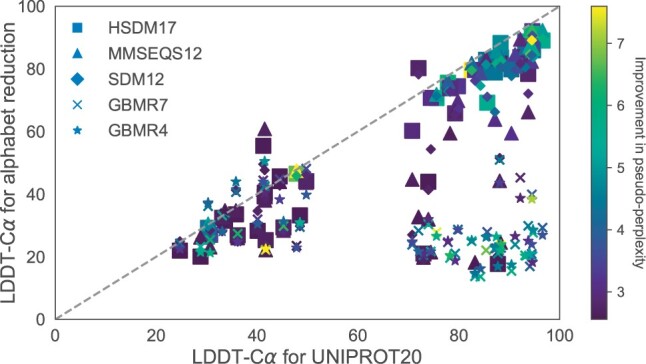
LDDT-Cα values for CAPS14 targets using different sequence encodings as input to ESMFold. The colour of each point represents the improvement in the pseudo-perplexity (reducing) compared to the full alphabet sequence. Small alphabets improve the pseudo-perplexity, but this is not directly related to improvements in LDDT-Cα. Targets that display higher LDDT-Cα scores do not improve the pseudo-perplexity by a large margin.

In Fig. 10, it can be observed that improving the pseudo-perplexity by means of alphabet reduction methods, does not entail more accurate predictions. For instance, despite small alphabets improving pseudo-perplexity, no corresponding enhancement in structural accuracy is observed. Targets that are better predicted using reduced alphabets only showcase a small improvement in pseudo-perplexity.

While both MSA depth and pseudo-perplexity values appear promising in providing explanations for the enhancements in LDDT-Cα scores, there is no straightforward explanation for the performance boost of ESMFold when utilizing reduced alphabets. Most of the targets from CASP14 show a performance degradation in terms of structure prediction accuracy when using reduced alphabets. This suggests that the model is simply able to generalize better for a subset of sequences when faced with a slightly modified input sequence. It could be the case that both the MSA and pseudo-perplexity are important factors, but the way this influences the modelling phase is not directly observable.

## 4 Conclusion

Multiple reduced amino acid alphabets have been proposed in the past but with the exception of those used in protein similarity comparison algorithms (Buchfink *et al.* 2015, Steinegger and Söding 2017), reduced alphabets are not applied to specific research work (Liang *et al.* 2022). Here, we explore the possibility of applying these reduced amino acid alphabets to PLMs.

Our findings suggest that PLM can still learn meaningful embeddings using reduced amino acid alphabets. However, they fail to consistently perform better on downstream tasks compared to the full alphabet. The beauty of training PLMs is that advantages in reduced alphabet representations previously sought by previous authors to eliminate complexity diminish in the field of unsupervised learning. The ability of PLM to learn a systematic understanding of amino acid properties with a large amount of data suggests that a predefined clustering scheme hinders the understanding of protein evolution. Although attractive as a means of making PLM more efficient in terms of dataset training size, reduced alphabets are unlikely to improve how language models learn the ’language of life’ (Nambiar *et al.* 2020)

However, reduced amino acid alphabets prove to be helpful in protein structure prediction. Namely, ESMfold performs better on 10 out of 50 CAPS14 targets when faced with a modified sequence as input. We believe this is due to reduced alphabets enabling the language model to artificially relate the input sequence to a larger number of possible structural matches seen during training. Considering the improvements brought to structural prediction, further use of alphabet reduction schemes could be used to model sequences with shallow MSAs when using single sequence folding models.

## Supplementary Material

btae061_Supplementary_Data

## Data Availability

The data is included in the GitHub repo.
