# Supplementary material for: Protein language models meet reduced amino acid alphabets
Source: Bioinformatics. 2024 Feb 3;40(2):btae061. doi: 10.1093/bioinformatics/btae061 (PMC10872054; doi:10.1093/bioinformatics/btae061)
Supplement: btae061_Supplementary_Data [file btae061_supplementary_data.pdf]

Bioinformatics
doi.10.1093/bioinformatics/xxxxxx
Advance Access Publication Date: Day Month Year
Manuscript Category

Supplementary material

Protein language models meet reduced amino acid alphabets

Ioan Ieremie [1,*], Rob M. Ewing [2,] and Mahesan Niranjan [1]

[1] Vision, Learning & Control Group, University of Southampton, Southampton, SO17 1BJ, UK and
[2] Biological Sciences, University of Southampton, Southampton, SO17 1BJ, UK.

Abstract

Motivation: Protein Language Models (PLMs), which borrowed ideas for modelling and inference from Natural Language Processing, have demonstrated the ability to extract meaningful representations in an unsupervised way. This led to significant performance improvement in several downstream tasks. Clustering amino acids based on their physical-chemical properties to achieve reduced alphabets has been of interest in past research, but their application to PLMs or folding models is unexplored.
Results: Here, we investigate the efficacy of PLMs trained on reduced amino acid alphabets in capturing evolutionary information, and we explore how the loss of protein sequence information impacts learned representations and downstream task performance. Our empirical work shows that PLMs trained on the full alphabet and a large number of sequences capture fine details that are lost in alphabet reduction methods. We further show the ability of a structure prediction model(ESMFold) to fold CASP14 protein sequences translated using a reduced alphabet. For 10 proteins out of the 50 targets, reduced alphabets improve structural predictions with LDDT-Cα differences of up to 19%.
Availability: Trained models and code are available at `github.com/Ieremie/reduced-alph-PLM`
Contact: ii1g17@soton.ac.uk

© The Author 2015. Published by Oxford University Press. All rights reserved. For permissions, please e-mail: journals.permissions@oup.com

1 The filtering of enzyme dataset

Upon further inspection of the Enzyme dataset from (Hermosilla Casajús *et al.*, 2021), we found a number of proteins that have the same sequence appearing between the dataset splits. Even if at the structure level there might be slight differences due to different experimental conditions, these are still the same proteins that can induce data leakage.

- PDB ids **5lf0_W, 5m32_I, 5le5_T, 5lf1_I, 5lf3_I, 5gjq_q** all point to the same Human proteasome complex, having the same protein sequence with **UniProtKB accession code: P49720**. These were found mixed between training and validation.
- PDB ids **3von_E, 3von_b, 3von_p, 3von_i** are part of the same protein complex, Crystal structure of the ubiquitin protease, having the same protein sequence with **UniProtKB accession: P61088**. These were found mixed between training and testing.
- PDB ids **3mg8_I, 4qlq_W, 6huv_I, 5fga_W, 4qby_W, 5mp_j** all point to the same Yeast proteasome complex, having the same protein sequence with **UniProtKB accession: P25451**. These were found mixed between training and testing.
- PDB ids **4y84_X, 5l5e_X, 6huu_J, 4qby_J, 4ya9_J, 5mp9_k, 5mpa_k** all point to the same Yeast proteasome complex, having the same protein sequence with **UniProtKB accession: P22141**. These were found mixed between training and testing.
- PDB ids **6hed_4, 6hec_5, 6he8_4, 6he9_3, 6he7_6, 6he8_k, 6hed_h, 6hea_i, 6hea_h, 6he9_i** all point to the same PAN-proteasome complex, having the same protein sequence with **UniProtKB accession: P25451**. These were found mixed between training and testing.
- PDB ids **5lf1_b, 5lf1_B, 5gjq_j** all point to the same Human proteasome complex, having the same protein sequence with **UniProtKB accession: P25789**. These were found mixed between training and testing.
- PDB ids **1iru_R, 5gjq_k** all point to the same Human proteasome complex, having the same protein sequence with **UniProtKB accession: Q3ZBG0**. These were found mixed between training and testing.

  Out of the 37428 data samples, there are only 15640 unique protein chains. This redundancy was kept in (Hermosilla Casajús *et al.*, 2021) due to working directly on structures which can be seen as a data augmentation, however, at the sequence level, this is not the case.

2 UNIPROT19 alphabets

Based on the clustering of the amino acids using the learned projections, we pretrain 3 other language models. These only combine two residues into a single token. We report the results on the downstream tasks in the following tables.

Alphabet	FOLD			REACT %
	Fold %	Super. %	Fam. %	
**UNIPROT20**	26.3 ± 0.96	43.3 ± 0.41	90.7 ± 0.44	81.8 ± 0.39
**UNIPROT19_P_E**	24.5 ± 0.57	41.7 ± 0.98	88.4 ± 1.00	80.4 ± 0.94
**UNIPROT19_V_S**	24.2 ± 0.46	38.5 ± 0.93	86.5 ± 1.59	77.9 ± 0.80
**UNIPROT19_L_H**	24.9 ± 0.28	40.2 ± 0.90	89.1 ± 0.44	80.4 ± 1.35

**Alphabet**	AAV				GB1			Meltome
	1-vs-many	2-vs-many	7-vs-many	low-vs-high	2-vs-many	3-vs-many	low-vs-high	mixed-split
**UNIPROT20**	0.41 ± 0.08	0.48 ± 0.00	0.55 ± 0.06	0.20 ± 0.04	0.64 ± 0.03	0.82 ± 0	0.39 ± 0.09	0.28
**UNIPROT19_P_E**	0.42 ± 0.03	0.51 ± 0.09	0.57 ± 0.04	0.20 ± 0.04	0.65 ± 0.02	0.82 ± 0	0.48 ± 0.03	0.26
**UNIPROT19_V_S**	0.42 ± 0.05	0.26 ± 0.28	0.62 ± 0.00	0.18 ± 0.05	0.63 ± 0.01	0.81 ± 0	0.40 ± 0.05	0.24
**UNIPROT19_L_H**	0.39 ± 0.03	0.59 ± 0.04	0.63 ± 0.00	0.16 ± 0	0.63 ± 0.04	0.81 ± 0	0.16 ± 0.09	0.27

3 Identity and similarity values for the translated CASP14 proteins

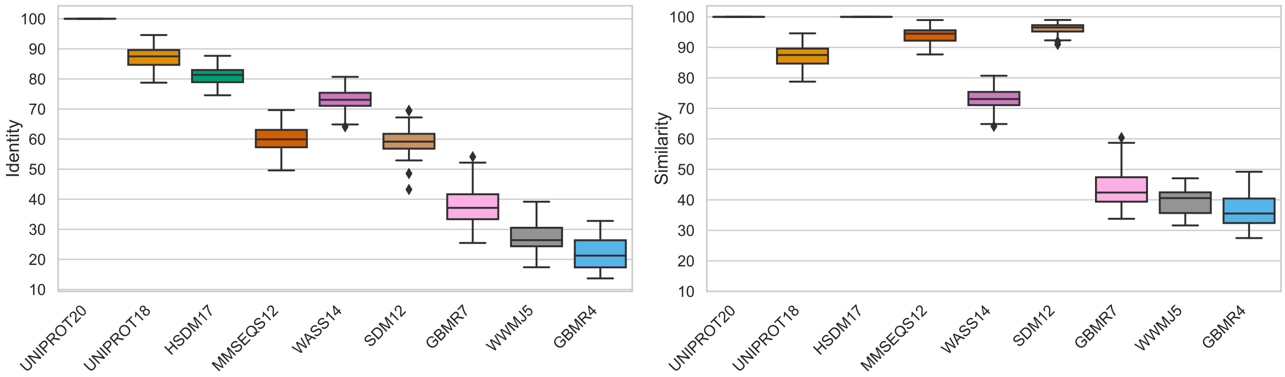

Fig. 1. The identity and similarity values between the translated proteins from CASP14 and the original full alphabet sequences. Sequences are aligned using a global pairwise algorithm, with a gap penalty of 10 and an extension gap penalty of 0.5. The similarity values are calculated using Blosum62. As alphabets get smaller in size, the identity between the modified sequence and the original sequence decreases. On the other hand, in terms of similarity, the alphabets HSDM17, MMSEQS13, and SDM12 maintain high similarity values. In particular, sequences modified using the HSDM17 alphabet all have 100% similarity to the original sequence. Alphabets UNIRPOT18 and WASS14 display the same similarity and identity values. This suggests that the clusters defined in their reduction scheme do not align with the information contained in Blosum62.

4 The determination of the WASS14 alphabet

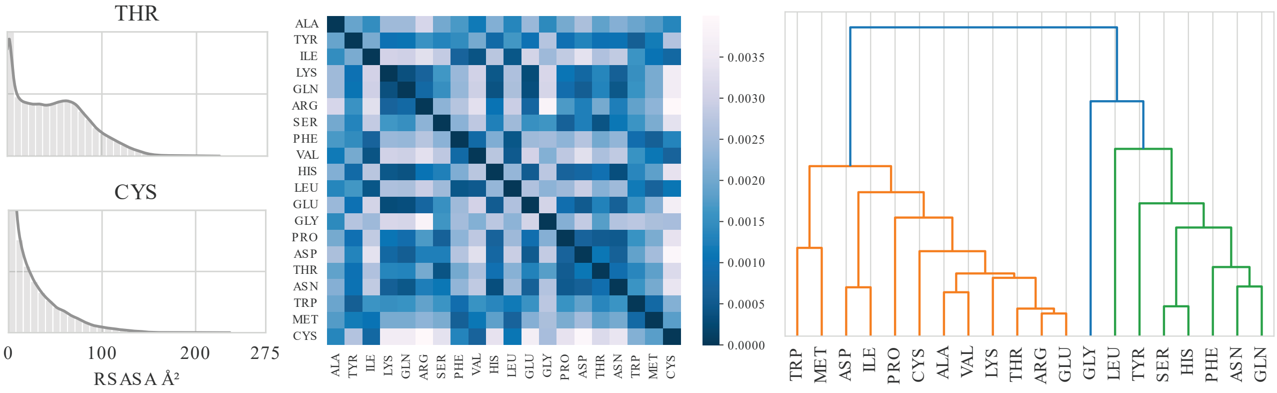

Fig. 2. Alphabet reduction method based on hydrophobicity profiles. For a set of proteins in the SCOPe database (version 2.06) (Chandonia et al., 2019), the solvent-accessible surface area is calculated for each amino acid type. An example of the RSASA distribution for residues Thr and Cys is shown on the left. Each RSASA distribution is then binned and a Wasserstein distance is computed between each amino acid pair. These values can be viewed as a similarity matrix (middle). Based on the similarity matrix, amino acids are clustered together and plotted using a dendrogram (right). The first amino acid pairs clustered together are then considered synonymous and encoded in the same way.

5 Selecting residues from the cluster at random along the sequence

Instead of choosing a random residue from the cluster and keeping it fixed throughout the sequence, we consider the option of selecting a residue randomly from the cluster for each position in the sequence.

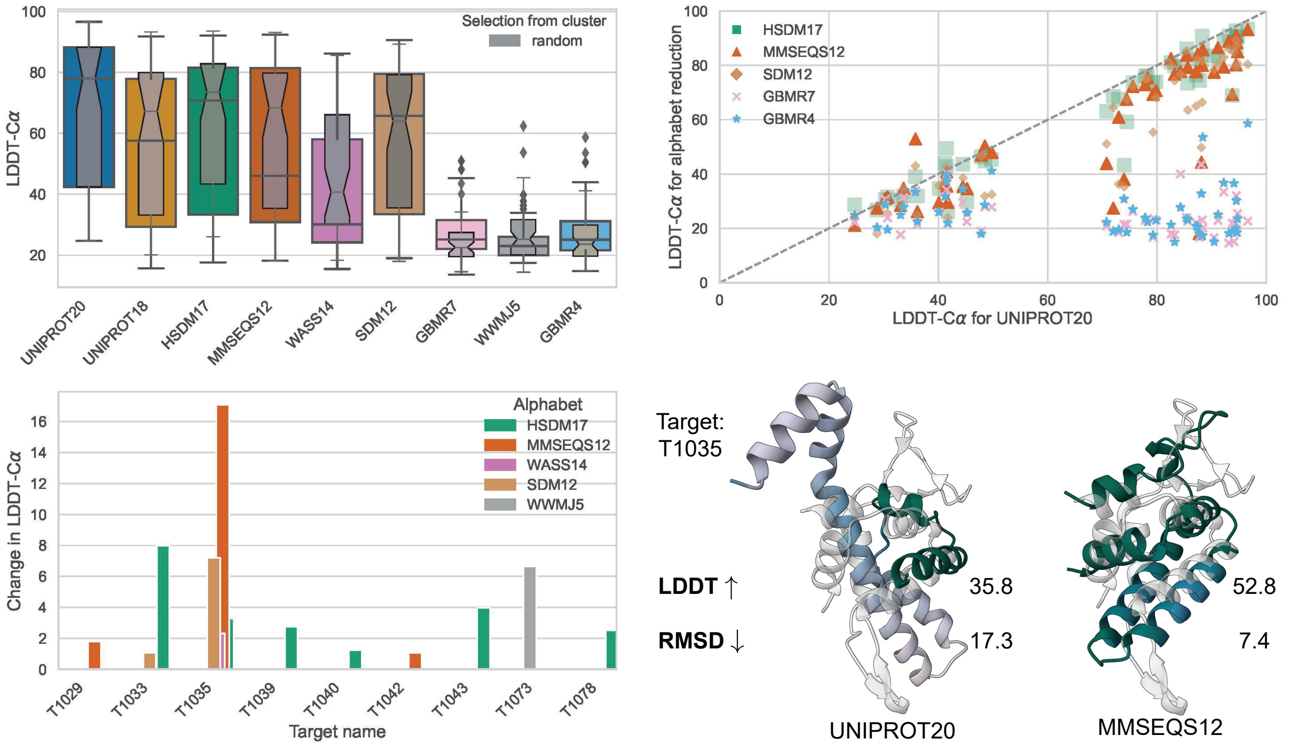

Fig. 3. Performance plots for reduced alphabets when residues are selected randomly from their cluster along the sequence. The overall performance for CAPS14 targets compared to the full alphabet is better when residues are chosen at random from the cluster at every position. However, the number of targets with high improvements (LDDT-Cα change > 5%) over the full alphabet predictions is smaller. The target T1035 has an improvement of 17% compared to the prediction that uses the full alphabet. This improvement is also higher than the GMBR4 performance when a single residue is used from each cluster and kept fixed.

6 Exploring all ways of translating sequences using a fixed residue

In the main document, we select a random amino acid from each cluster and keep it fixed along the sequence. However, there are many other ways of selecting such residues and generating different sequences. In this section, we look at all possible ways of selecting a representative residue from a cluster and keeping it fixed along the sequence. Generating all possible combinations for the 51 proteins from CASP14 and each alphabet, we end up with 49292 translated sequences. We predict the structures for each sequence using ESMfold and analyse the results in Figure 4.

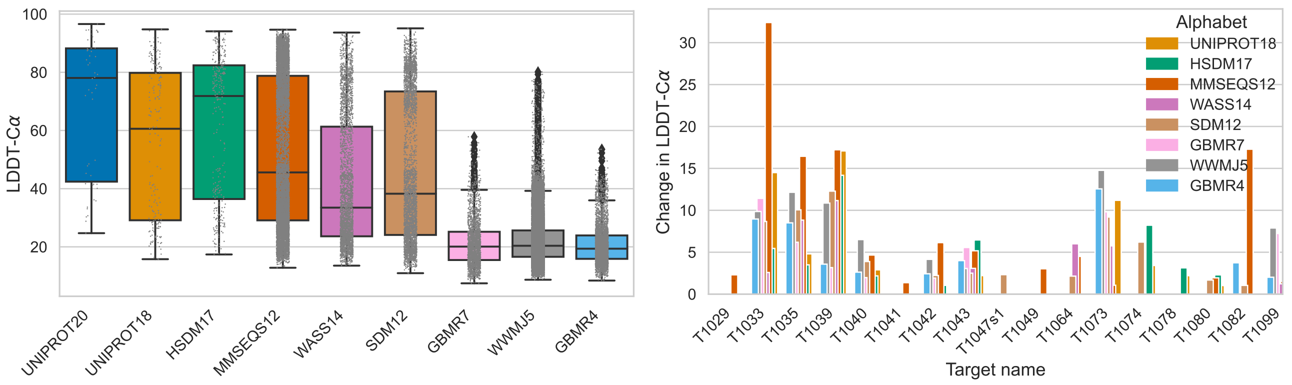

Fig. 4. On the left the LDDT-Cα for each alphabet from all possible combinations is plotted as a box plot. On the right, we select only those proteins for which the structure prediction improves the LDDT-Cα metric. A higher number of proteins get structural prediction improvements compared to exploring a single way of translating the protein sequence (see the main document).

References

Chandonia, J.-M. *et al.* (2019). Scope: classification of large macromolecular structures in the structural classification of proteins—extended database. *Nucleic acids research*, **47**(D1), D475–D481.

Hermosilla Casajús, P. *et al.* (2021). Intrinsic-extrinsic convolution and pooling for learning on 3d protein structures. In *International Conference on Learning Representations, ICLR 2021: Vienna, Austria, May 04 2021*, pages 1–16. OpenReview. net.
